# Validation of Reference Genes for Gene Expression by Quantitative Real-Time RT-PCR in Stem Segments Spanning Primary to Secondary Growth in *Populus tomentosa*

**DOI:** 10.1371/journal.pone.0157370

**Published:** 2016-06-14

**Authors:** Ying Wang, Yajuan Chen, Liping Ding, Jiewei Zhang, Jianhua Wei, Hongzhi Wang

**Affiliations:** Beijing Key Laboratory of Agriculture Gene Resource and Biotechnology, Beijing Academy of Agriculture and Forestry Sciences, Beijing, P. R. China; University of New England, AUSTRALIA

## Abstract

The vertical segments of *Populus* stems are an ideal experimental system for analyzing the gene expression patterns involved in primary and secondary growth during wood formation. Suitable internal control genes are indispensable to quantitative real time PCR (qRT-PCR) assays of gene expression. In this study, the expression stability of eight candidate reference genes was evaluated in a series of vertical stem segments of *Populus tomentosa*. Analysis through software packages geNorm, NormFinder and BestKeeper showed that genes ribosomal protein (*RP*) and tubulin beta (*TUBB*) were the most unstable across the developmental stages of *P*. *tomentosa* stems, and the combination of the three reference genes, eukaryotic translation initiation factor 5A (*eIF5A*), Actin (*ACT6*) and elongation factor 1-beta (*EF1-beta*) can provide accurate and reliable normalization of qRT–PCR analysis for target gene expression in stem segments undergoing primary and secondary growth in *P*. *tomentosa*. These results provide crucial information for transcriptional analysis in the *P*. *tomentosa* stem, which may help to improve the quality of gene expression data in these vertical stem segments, which constitute an excellent plant system for the study of wood formation.

## Introduction

Gene expression analysis always provides important clues regarding gene function in plant growth and development and response to environmental changes. Currently available methods of assessing gene expression include RNA *in situ* hybridization, Northern blotting, expression pattern of report genes driven by the promoter of the specific gene, RNase protection assay, microarray analysis, transcriptome sequencing, and quantitative real time PCR (qRT-PCR). qRT-PCR is considered the most powerful and efficient technique for quantifying levels of gene transcription due to its high sensitivity, reproducibility, safety, and simple operation. Reference genes with stable expression levels are indispensable to normalizing the expression levels of the target genes in qRT-PCR analysis in order to control the variation caused by the differences in initial RNA amount, RNA integrity and purity, and enzyme efficiency [1, 2].

There are several housekeeping genes, which are involved in basic cellular and metabolic processes that are usually used as reference genes in qRT-PCR assays. These include ribosomal protein (*RP*), tubulin (*TUB*), actin (*ACT*), 18S rRNA (*18S*), elongation factor (*EF*), eukaryotic translation initiation factor (*eIF*), ubiquitin (*UBQ*), and histone H3 (*HIS3*) [3–6]. It is assumed that these genes present constant and stable expression across different developmental stages, treatments, and environmental conditions. However, most of the commonly used housekeeping genes were shown to be highly variable, and using them as reference genes can produce inaccurate expression patterns [2, 7–9]. It is usually not practical to find an ideal universal reference gene that is expressed constantly and stably in many types of tissues under highly different conditions. Validation of reference genes is highly specific to particular experimental models, and it is a crucial step in the initiation of analysis of RNA transcription levels by qRT-PCR in a series of specific tissues with a given developmental period [2, 6, 7, 9, 10].

Several software packages have been developed to identify reference genes with stable expression levels, such as geNorm [11], NormFinder [12], and BestKeeper [13]. With the help of these statistical algorithms, a number of studies on validation of reference genes in plant have recently been reported. In soybeans, SKP1/Ask-Interacting Protein 16, *UKN1* (a hypothetical protein) and metalloprotease have been shown to be the suitable for different developmental stages, whereas *Actin II*, alpha tubulin, and *TIP41*-like family protein in varied photoperiodic treatments [14]. In peaches, translation elongation factor 2, RNA polymerase II, and ubiquitin 10 showed remarkably stable expression levels and were always classified among the top four positions of the ten test reference genes in the varied series of samples, including different stages of fruit development, different genotypes, different storage time series, different exogenous regulator treatments, and different tissues [15]. In chicory, actin, elongation factor 1-alpha, and 18S rRNA had the highest expression stability across leaf and root tissues [16]. In strawberries, a DNA binding protein (*DBP*) was the most suitable reference gene in samples of different cultivars and under drought stress conditions, and histone *H4* under osmotic stresses and salt stress [9]. All results indicated that the expression stability of reference gene is dependent on the experimental conditions and developmental stages [9, 17–19].

Wood is the most abundant biomass produced by land plants, considered to be one of the most renewable sources of bioenergy [20]. As a model plant of wood formation and differentiation, *Populus* is widely used for the study of deciphering the biosynthesis and regulatory mechanisms of secondary cell walls during wood formation. Cell wall thickening and programmed cell death are the main developmental events in secondary growth, during which the molecular process were extensively reported in the past few years [21–25]. However, the developmental events involved in the transition from primary to secondary growth are not well known. The terminal region of developing *Populus* stems represent different developmental phases from primary to secondary growth. These provide an experimental system for elucidating the physiological and molecular processes specific to secondary growth in wood formation. To date, there have been no reports on the choice of suitable qRT-PCR internal control genes used for gene expression studies in *Populus* stem segments spanning from primary to secondary growth. For this reason, in this study, eight reference genes involved in different biological functions, including the formation of cellular cytoskeleton (actin and tubulin beta), initiation phase of translation (eukaryotic translation initiation factor 5A), elongation phase of translation (elongation factor 1-beta), DNA packaging (histone H3), protein biosynthesis (ribosomal protein and 18S ribosomal RNA), and protein degradation (ubiquitin protein), were selected for analysis of the stability of expression in *Populus tomentosa* stem segments across several stages of development using three software packages geNorm, NormFinder, and BestKeeper.

## Materials and Methods

### Plant material

White poplar clone (*P*. *tomentosa* Carr. *cv* ‘BJHR01’), grown in a growth camber at the Beijing Academy of Agriculture and Forestry Sciences, was used for the experiments. No specific permits were required by the scientific research institute to use these materials. Clonal propagation were conducted by excising nodal segments and allowing axillary buds to elongate and roots to regenerate through tissue culture as described by Lu et al. [26]. Plants were then cultivated in pots with commercial soil in a greenhouse at the Beijing Academy of Agriculture and Forestry Sciences, Beijing, and grown under a 12 h/12 h day/night photoperiod at 25°C. When the trees reached 2 months of age, i.e. plastochron index about 51 [27], the following samples were harvested from the stem: segments with the leaf plastochron indexes (LPI) 2, 3, 5, and 9 [27]. All samples were quickly frozen in liquid nitrogen and stored at -80°C. Six trees were used in this analysis.

### Total RNA extraction

Total RNA was isolated from stem segments LPI 2, 3, 5, and 9 of white poplar using the RNeasy Plant Mini Kit (Cat. No. 74903, Qiagen) according to the manufacturer’s instructions. On-column DNase digestion was performed using the RNase-free-DNase set (Cat. No. 79254, Qiagen). The integrity of RNA samples was assessed using gel electrophoresis, and purity was assessed with 260/280 and 260/230 ratios. RNA concentration was determined using a NanoDrop 2000 c spectrophotometer (Thermo Scientific).

### RT-qPCR

Eight housekeeping genes were selected to identify the most stably expressed reference genes in *P*. *tomentosa* stem segments across different stages of development, including primary growth, transition from primary growth to secondary growth, and secondary growth. Gene sequences were obtained from *Populus trichocarpa* genome annotation v3.0 (http://phytozome.jgi.doe.gov/pz/portal.html), with an exception of the sequence of 18S ribosomal RNA from the report by Brentner [28]. qRT-PCR primers were designed using Primer3 (http://bioinfo.ut.ee/primer3-0.4.0/). Each primer set was expected to amplify a PCR product with a length of 80–150 bp. For each targeted gene, 3 sets of primers were prepared, and the specificity of all primers was checked by BLAST against the *P*. *trichocarpa* genome. The best set of primers, was identified based on sequence of PCR product and dissociation curve analysis and PCR amplification efficiency.

cDNA was synthesized by using a SuperScript® III First-strand (Invitrogen, Cat. No. 18080–051) with 1 μg total RNA as template. Real-time PCRs were conducted in Bio-Rad Real-Time System (CRX96™ Real-Time System). For each reaction, the 20 μL mixture contained the first-strand cDNA (equivalent to 100 ng of total RNA), 5 pmol each of the forward and reverse primers, and 10 μL of SYBR Green PCR master mix (2 X SYBR Green PCR master mix, TakaRa SYBR® Premix Ex Taq™, Cat. No. RR4320A). The amplification program was as follows: 95°C for 30 s, 40 cycles at 95°C for 5 s, 58°C for 15 s, and 72°C for 30 s. After amplification, a thermal denaturing cycle was added to derive the dissociation curve of the PCR product to verify amplification specificity. Each reaction was repeated at least three times.

### Data analysis

The LinRegPCR 2014.x program was used to calculate the mean amplification efficiencies and coefficient of correlation for each primer set [29]. The descriptive statistics of the expression levels were computed for each candidate reference gene using the software package BestKeeper. Quantification cycle (Cq) values were converted to relative expression quantities using Relative Expression Software [30], and the latter values were required by software geNorm and NormFinder. The expression stability of the eight housekeeping genes in *P*. *tomentosa* stem segments with primary or secondary growth was assessed by using software geNorm, NormFinder, and BestKeeper.

#### geNorm

geNorm is a statistical algorithm which relies on the principle that the expression ratio of two ideal internal control genes is identical in all tested samples, regardless of the experimental condition or tissue type [11]. For a particular candidate reference gene, the gene-stablity measure *M* is defined as the average pairwise variation with all other tested control genes. Lower *M* values indicate more stable expression patterns. Stepwise exclusion of the gene with the highest *M* value and recalculation of new *M* values for the remaining genes allows ranking of the tested genes according to their expression stability, and results in a combination of two constitutively expressed internal control genes that have the most stable expression in all the tested samples. The geNorm program also calculates the normalization factor for accurate normalization, which is the geometric mean of the selected most stable reference genes, by stepwise inclusion of a less stable gene until the (n+1)^th^ gene has no significant contribution to the newly calculated normalization factor NF_n+1_ [11]. Particularly, if the pairwise variation V_n/n+1_ between the two sequential normalization factors NF_n_ and NF_n+1_ is lower than the cutoff value of 0.15, this suggested the inclusion of the added control gene is not required.

#### NormFinder

NormFinder uses a strategy rooted in a mathematical model of gene expression to identify stably expressed genes among a number of candidate reference genes. It takes into account intra- and intergroup variations in defined samples groups. The stability value for each candidate reference gene is calculated by combining intra- and intergroup variations of gene expression [12]. The most stably expressed gene is the one with the lowest expression stability values, and the lowest variation values within and between the groups.

#### BestKeeper

BestKeeper ranks the candidate reference genes based on gene Cq variation and correlation with the BestKeeper index value [13]. Higher variation, which is displayed as standard deviation (SD), indicates more unstable expression pattern. The SD threshold value of 1.0 is suggested by Pfaffl et al. [13], and candidate genes with SD values greater than 1 are considered unstable. To calculate the BestKeeper index value, the pairwise correlation of all possible candidate reference genes is estimated by calculating Pearson correlation coefficient (*r*) and the probability *P* value. The selected stably expressed inter control genes with lower SD and high inter-gene correlation are combined into the BestKeeper index, as calculated as the geometric mean of Cq values of the selected genes. The genes highly correlated with the BestKeeper index are recommended as stable. BestKeeper is also used to test the sample integrity (e.g. mRNA respectively cDNA quantity and quality) by calculating an intrinsic variance (InVar) of Cq, which is expressed as an efficiency corrected intrinsic variation of x-fold. A sample should be removed due to poor template integrity when this value is over a 3-fold over- or under-expression.

### Plant stem sectioning and microscopy

Fresh stem segments of each internode were hand-sectioned using double-edged razor blade. The sections were stained with 0.05% aqueous Toluidine Blue-O dye shortly, rinsed in water, and examined under bright-field microscopy. Toluidine Blue-O stains lignified walls bright blue, and most primary (non-lignified) walls purple.

## Results

### Vertical stem segments represent different developmental stages

**Fig 1:**
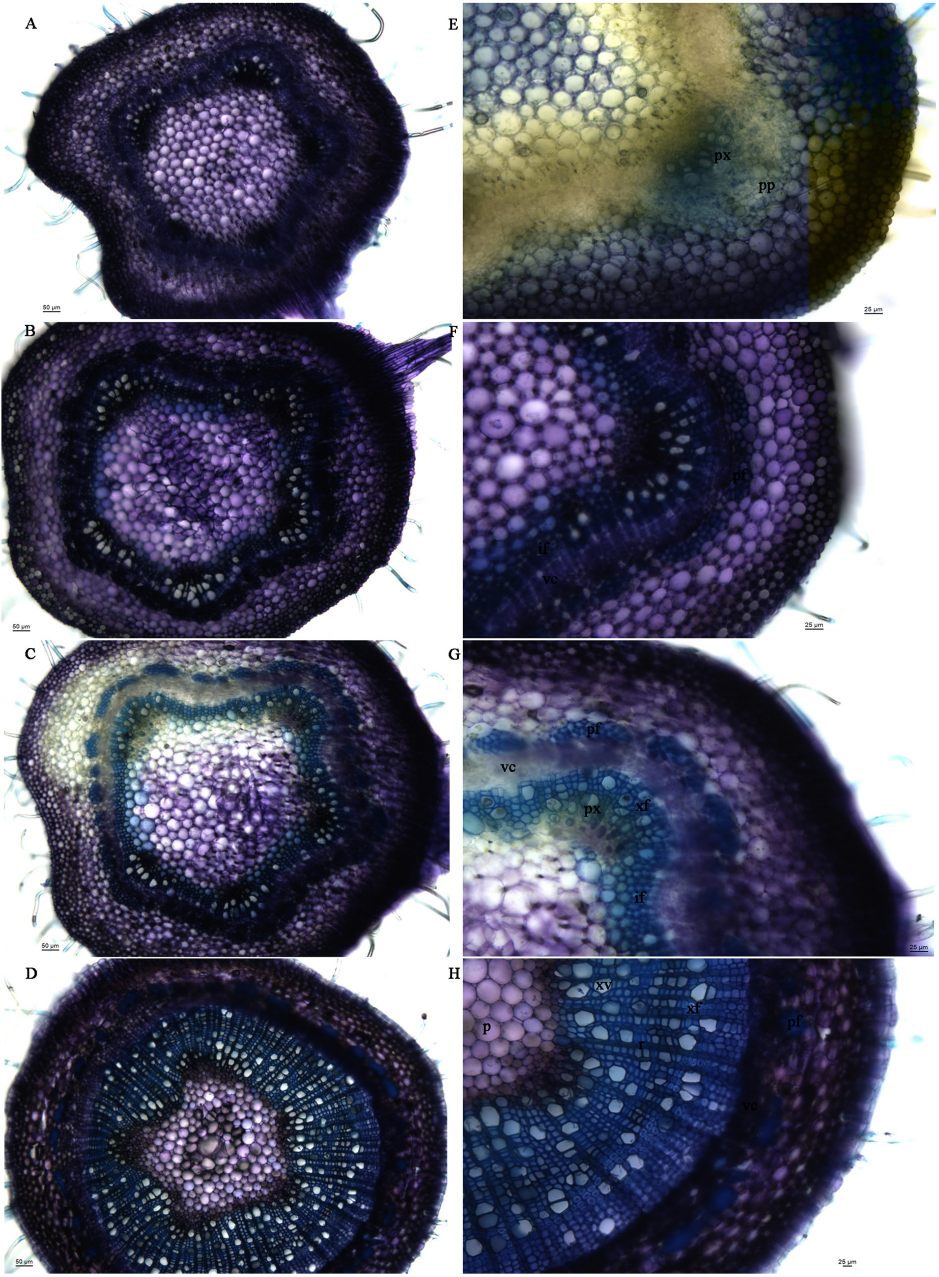
Sectioning of stem segments from 2-month-old white poplar trees. Four stem segments were collected at the following points: 1 cm below the apical meristem and at LPI 4, 5, and 9, respectively. Hand-made transverse sections were stained with 0.05% Toluidine Blue-O. A, E: The stem segment 1 cm below the apical meristem, showing the primary xylem (px) and primary phloem tissues (pp) from procambial cells; B, F: The stem segment LPI 4 with formation of secondary vascular cambium ring (vc), xylem interfascicular fiber (if) and wall–thickened phloem fiber (pf); C, G: The stem segment LPI 5, where secondary xylem grew rapidly with the concurrence of xylem fiber cells (xf); D, H: The stem segment LPI 9 with abundant secondary xylem, including xylem vessel (xv), xylem fiber (xf), ray (r), and pith (p).

Transverse sections of stem segments from two-month-old trees were investigated to determine developmental stages (Fig 1). The stem segments adjacent the apical meristem showed primary growth (Fig 1A and 1E), where primary xylem and phloem tissues were formed from procambial cells. The primary growth in the top stem regions (above internode 4) contributed mainly to stem elongation. Secondary growth initiated in the regions LPI 4 and 5, where the secondary vascular cambium ring and xylem interfascicular cells had emerged, and the cell walls of phloem fiber had thickened (Fig 1B, 1C, 1F and 1G). In stem internodes below the regions LPI 5, the amount of secondary xylem increased rapidly, and fiber cells were heavily lignified, and wood formed (Fig 1D and 1H). In conclusion, the vertical segments of *P*. *tomentosa* stem represent different developmental stages involved in wood formation, including primary growth, transitional process from primary and secondary growth, and secondary growth.

### Primer specificity and expression levels of candidate reference genes in different internodes

**Fig 2:**
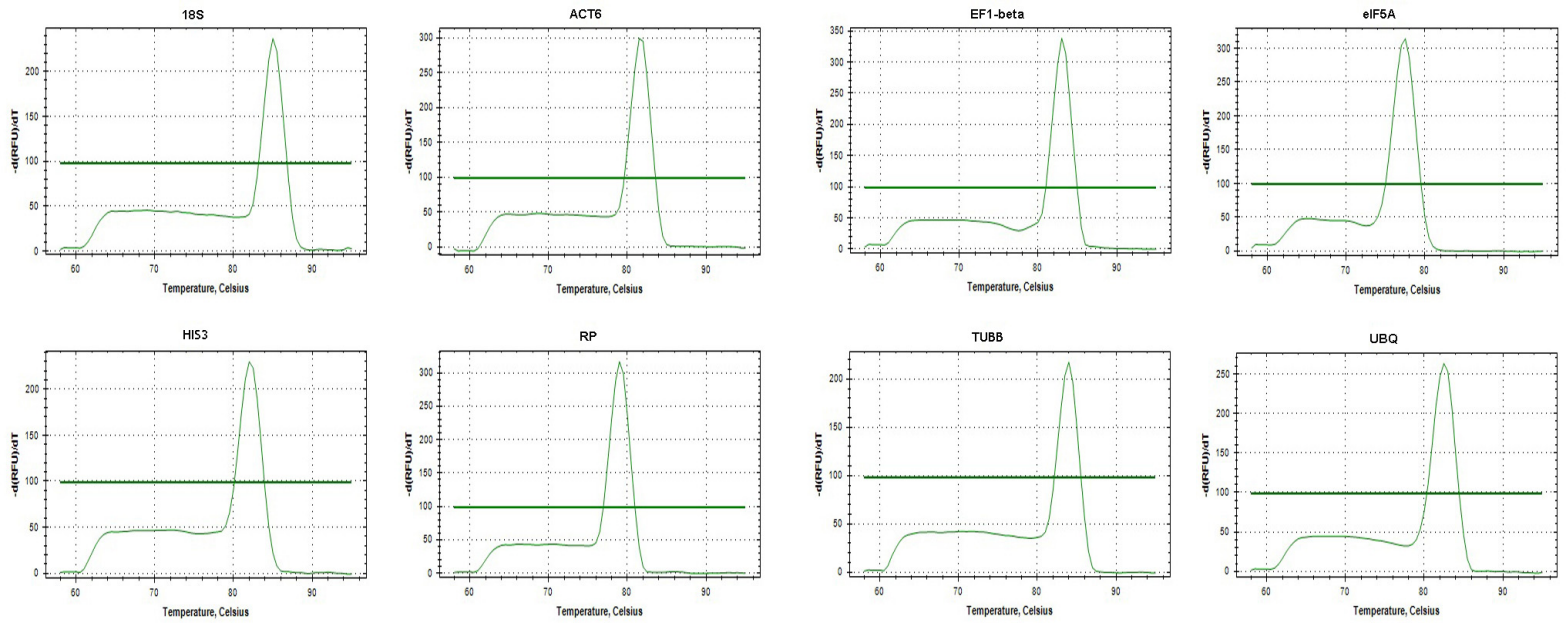
Specificity of qRT-PCR amplification. Melting curves of the 8 amplicons (*18S*, *ACT6*, *EF1-beta*, *eIF5A*, *HIS3*, *RP*, *TUBB*, *UBQ*), all showing single peak.

Primers used to evaluate the expression stability of the candidate reference genes in different internodes were the ones with the best performance in specificity and amplification efficiency (Table 1). Mean amplification efficiency (E) varied from 90.3% for *ACT6* to 104.9% for *EF1-beta* and correlation coefficients (R^2^) of linear amplification was between 0.987–0.998. The specificity of the chosen primer was confirmed with a single peak in the melting curves (Fig 2).

**Table 1:** Descriptions of candidate reference genes and primer sequences for qRT–PCR.

Gene Name	Gene ID	Primer sequence (5′–3′)	Tentative annotation	E* (%)	R^2^**
***ACT6***	Potri.001G309500	AAACTGTAATGGTCCTCCCTCCG GCATCATCACAATCACTCTCCGA	Actin	90.3	0.996
***EF1-beta***	Potri.009G018600	AACCTGGTCGTGATTTCCCT ATCACCAGCAGCCTCCTTG	Elongation factor 1-beta	104.9	0.996
***UBQ***	Potri.014G115100	CCTTTGTGCTTGATGTGGGC TAAAGCCAGGAGATGCAGCC	Ubiquitin protein	104.8	0.996
***RP***	Potri.001G342500	GCAAGAGGGATTTTGGGAGC ACTTGGGTGGCCAGAACAAA	Ribosomal protein	103.4	0.997
***HIS3***	Potri.005G072300	TTTAAGACTGATCTGCGTTTCC GAACAGCCCAACAAGGTATG	Histone H3	102.7	0.996
***eIF5A***	Potri.018G107300	TCGTTCCTTCATCTCACAACTGT AGACTCACAAAGCCATCTTCAGA	Eukaryotic translation initiation factor 5A	101.4	0.987
***TUBB***	Potri.003G126800	AGCTGGCCGTGAACCTAATC CAGCTCAGGGATGGTCAAGG	Tubulin beta	100.0	0.998
***18S***	AY652861.1	CGAGACCTCAGCCTGCTAAC TGCGGCCCAGAACATCTAAG	18S ribosomal RNA	101.3	0.998

*E = mean PCR amplification efficiencies

** R^2^ = correlation coefficient

A total of 22 samples were analyzed to identify the most stable reference genes, with the exclusion of 2 samples due to poor RNA integrity (data not shown). The expression level displayed significant variation among reference genes, with Cq values ranging from 2.67 to 26.65 (Table 2). 18S showed the most abundant transcription level, reaching mean threshold fluorescence as few as 2.67 amplification cycles, while *RP* was the least abundant, needing as many as 26.65 amplification cycles to reach the mean threshold fluorescence. For a specific reference gene, the expression level varied among samples. For instance, the transcript abundance of gene *RP* changed dramatically with Cq values ranging from 15.42 to 26.65, while the genes *eIF5A*, *EF1-beta*, *HIS3*, and *ACT6*, showed stable expression level among samples, with SD much lower than 1 (Table 2).

**Table 2:** Descriptive statistics of eight candidate reference genes based on their Cq values.

Factor*	*18S*	*ACT6*	*EF1-beta*	*eIF5A*	*HIS3*	*RP*	*TUBB*	*UBQ*	BestKeeper (n = 8)
**N**	22	22	22	22	22	22	22	22	22
**GM[Cq]**	5.10	16.61	17.41	16.75	17.56	23.84	20.96	23.43	16.34
**AM[Cq]**	5.24	16.63	17.43	16.76	17.57	24.02	21.00	23.45	16.36
**Min[Cq]**	**2.67**	15.69	16.27	15.82	16.70	15.42	19.06	21.35	14.36
**Max[Cq]**	6.47	18.85	19.18	18.21	19.46	**26.65**	23.95	25.18	17.53
**SD[±Cq]**	0.93	0.59	0.49	0.47	0.55	**1.91**	**1.13**	0.85	0.63
**C**V**[%Cq]**	17.69	3.52	2.82	2.83	3.15	7.96	5.39	3.62	3.87

*N: number of samples; GM [Cq]: the geometric mean of Cq; AM [Cq]: the arithmetic mean of Cq; Min [Cq] and Max [Cq]: the extreme value of Cq; SD [±Cq]: the standard deviation of the Cq; CV [%Cq]: the coefficient of variance expressed as a percentage on the Cq level.

### Stability assessment of reference genes

The ranking of the eight candidate reference genes according to their expression stability across the stem segments at different developmental stages as calculated by geNorm, NormFinder and BestKeeper is shown in Table 3.

**Table 3:** Control genes ranked in order of their expression stability by geNorm, NormFinder and BestKeeper*.

geNorm	NormFinder	BestKeeper
*RP*	*TUBB*	*RP*
*TUBB*	*RP*	*TUBB*
*UBQ*	*UBQ*	*18S*
*18S*	*18S*	*UBQ*
*HIS3*	*eIF5A*	*ACT6*
*eIF5A*	*ACT6*	*HIS3*
*ACT6* and *EF1-beta*	*EF1-beta*	*EF1-beta*
	*HIS3*	*eIF5A*

*The expression stability increasing from top to bottom; the two most stable control genes, *ACT6* and *EF1-beta* as calculated by geNorm, can not be ranked in order because of the required use of gene ratios for gene-stability measurements.

**Fig 3:**
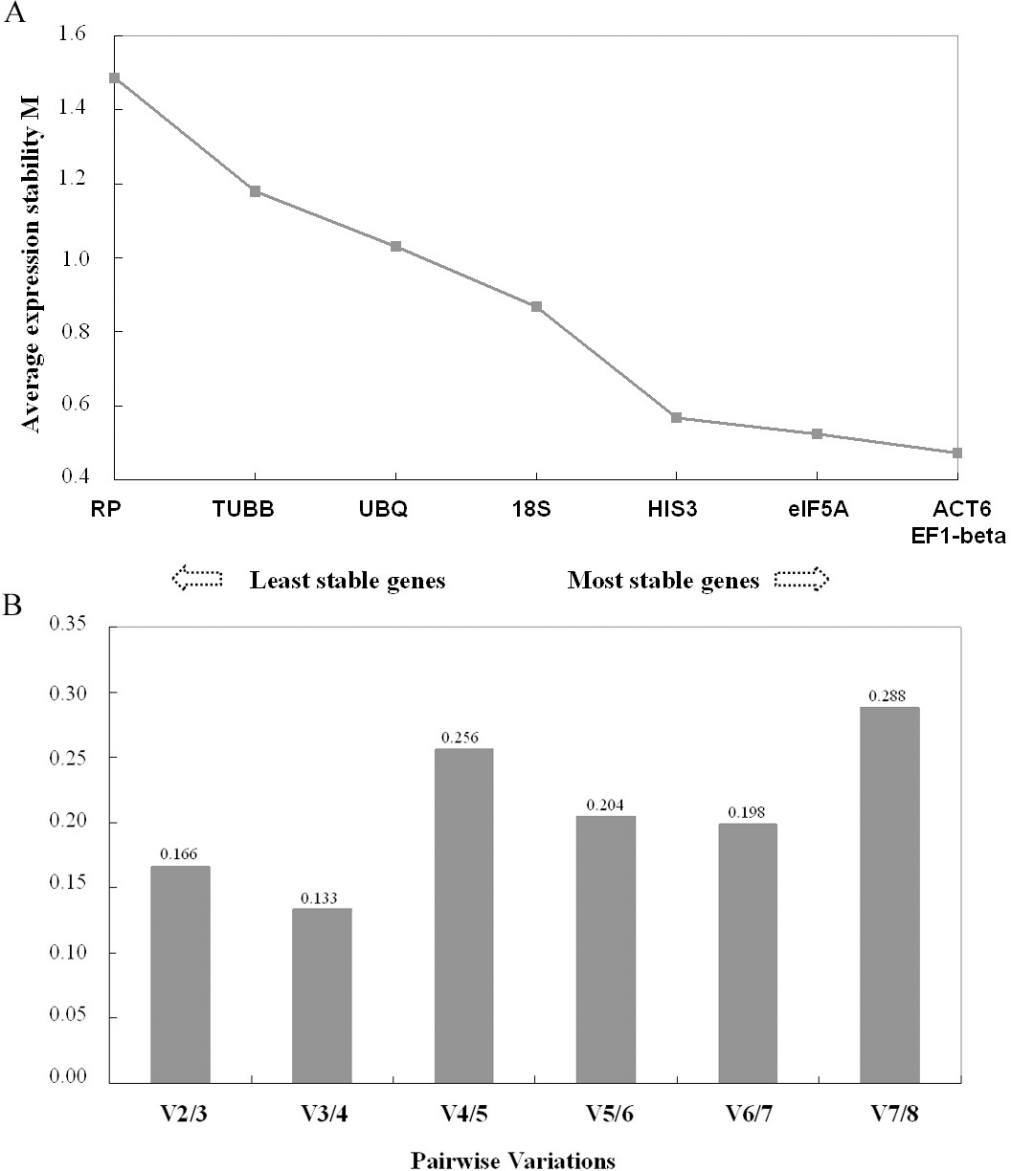
Validation of candidate reference genes by geNorm. A) Average expression stability values of remaining control genes plotted from least stable (left) to most stable (right). The average expression stability values were calculated for the remaining control genes after the least stable gene with the highest *M* value was excluded from the new calculation round. B) Pairwise variation (V) analysis to determine the optimal number of reference genes. The pairwise variation V_n/n+1_ was calculated between two sequential normalization factors NF_n_ and NF_n+1_.

geNorm ranks the tested genes from the least to the most stable according to the stability value *M* as follows: *RP* > *TUBB* > *UBQ* >*18S* > *HIS3* > *eIF5A* > ACT6 = EF1-beta (Table 3). With a *M* value around 0.5, *EF1-beta*, *ACT6*, *eIF5A*, and *HIS3* were found to be the most stable genes, as reported by Silveira et al. [31] and Yan et al. [32] (Fig 3A). Furthermore, the pairwise variation V_3/4_ between the two sequential normalization factor NF_3_ and NF_4_ was the lowest and smaller than the cutoff threshold of 0.15, as recommended by Vandesompele et al. [11], indicating that the combination of the three most stable genes (*ACT6*, *EF1-beta* and *eIF5A*) would be optimal for calculating the normalization factor and would give the accurate qRT–PCR normalization during stem development (Fig 3B).

**Fig 4:**
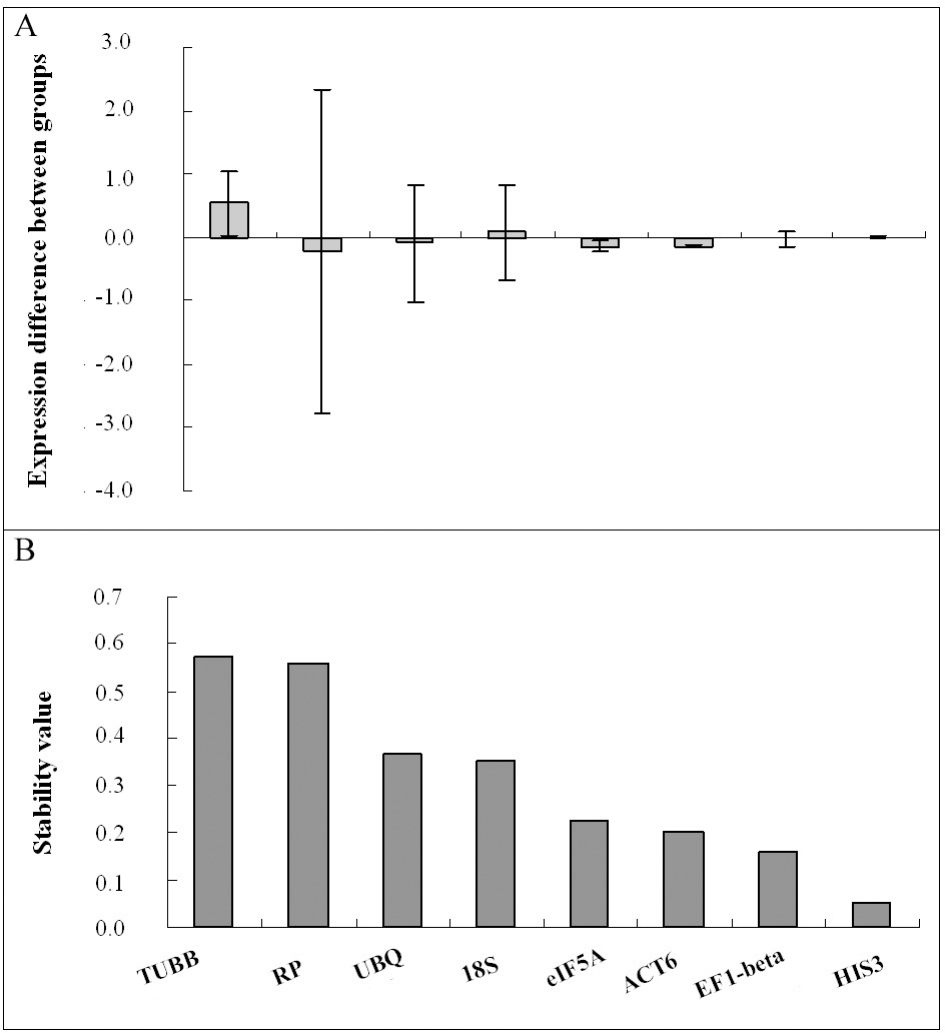
Gene expression stability of the candidate genes analyzed by NormFinder. A) Gene expression difference between two groups. Gene expression quantities were transformed from the Cq values by using Relative Expression Software [30]. Vertical bars represent the intragroup variation. B) Stability value of the candidate reference genes, which were ranked according to the value. The most stable reference gene had the lowest stability value, and the least stable had the highest value.

According to the program NormFinder, the experimental biological samples were divided into two groups based on their developmental stages. The two stem segments IN2 and IN3, representing primary growth, were considered an experimental group, and IN 5 and IN 9, with dominant secondary growth, were considered the other group. NormFinder ranks the candidate reference genes according to the stability values as follows: *TUBB* > *RP* > *UBQ* >*18S* > *eIF5A* > *ACT6* > *EF1-beta* > *HIS3* (Table 3). With the lowest stability values, between 0.052 and 0.224, reference genes *HIS3*, *EF1-beta*, *ACT6*, and *eIF5A* presented stable expression levels through *P*. *tomentosa* stem segments at different developmental stages (Fig 4). The stability values shown in Fig 4B are consistent with intra- and intergroup variations shown in Fig 4A.

Based on the Cq variation, BestKeeper preliminarily ranks the candidate reference genes as displayed in Table 3. *RP* and *TUBB* with the SD values 1.91 and 1.13, respectively, were excluded from the stably expressed genes, while *eIF5A*, *EF1-beta*, *HIS3*, and *ACT6* were among the most stable genes with the lowest SD value around 0.5–0.6 (Table 2). Pairwise correlation of the eight housekeeping genes, and correlation with the BestKeeper index were calculated to further rank the candidate reference genes. *eIF5A*, *ACT6*, and *EF1-beta* are here recommended as the most stable genes with a pairwise correlation coefficient of 0.643–0.739 (*P* = 0.001, Table 4) and correlation coefficient with the BestKeeper index of 0.879–0.909 (n = 3, the three selected stable reference genes, *eIF5A*, *ACT6*, and *EF1-beta*, used for calculating the BestKeeper index value, *P* = 0.001, see Table 5). *HIS3*, *18S*, and *UBQ* are excluded from the most stable genes with the poor correlation with other candidate genes and/or the BestKeeper index, although all of them had SD < 1 (Tables 2 and 4).

**Table 4:** Pairwise correlation analysis of the eight reference genes, and correlation analysis of the candidate reference genes with the BestKeeper index (n = 8) *.

**vs.**	***18S***	***ACT6***	***EF1-beta***	***eIF5A***	***HIS3***	***RP***	***TUBB***	***UBQ***
***ACT6***	0.298	-	-	-	-	-	-	-
***P*-value**	0.177	-	-	-	-	-	-	-
***EF1-beta***	0.656	**0.643**	-	-	-	-	-	-
***P*-value**	0.001	**0.001**	-	-	-	-	-	-
***eIF5A***	0.372	**0.739**	**0.729**	-	-	-	-	-
***P*-value**	0.089	**0.001**	**0.001**	-	-	-	-	-
***HIS3***	0.012	0.56	0.238	0.479	-	-	-	-
***P*-value**	0.961	0.007	0.284	0.024	-	-	-	-
***RP***	0.06	0.004	-0.097	0.1	0.319	-	-	-
***P*-value**	0.79	0.984	0.665	0.658	0.147	-	-	-
***TUBB***	0.268	0.081	0.318	0.259	-0.049	-0.274	-	-
***P*-value**	0.229	0.723	0.149	0.244	0.828	0.219	-	-
***UBQ***	0.325	0.492	0.508	0.398	0.505	0.218	-0.042	-
***P*-value**	0.139	0.02	0.016	0.067	0.016	0.329	0.851	-
**BestKeeper vs.**	***18S***	***ACT6***	***EF1-beta***	***eIF5A***	***HIS3***	***RP***	***TUBB***	***UBQ***
**Coeff. of corr. [*r*]**	0.843	0.561	0.75	0.646	0.401	0.391	0.278	0.585
***P*-value**	0.001	**0.007**	**0.001**	**0.001**	0.064	0.072	0.212	0.004

*Pearson correlation coefficient (*r*) and the value of probability *P* are shown

**Table 5:** Pairwise correlation analysis of the selected three reference genes, and correlation analysis of these genes with the BestKeeper index (n = 3) *.

**vs.**	***ACT6***	***EF1-beta***	***eIF5A***
***EF1-beta***	0.643	-	-
***P*-value**	0.001	-	-
***eIF5A***	0.739	0.729	-
***P*-value**	0.001	0.001	-
**BestKeeper vs.**	***ACT6***	***EF1-beta***	***eIF5A***
**Coeff. of corr. [*r*]**	0.898	0.879	0.909
***P*-value**	0.001	0.001	0.001

*Pearson correlation coefficient (*r*) and the value of probability *P* are shown.

## Discussion

In given species and tissues under specific experimental, it is crucial to validate the expression stability of reference genes for an accurate analysis of RNA transcription level by qRT-PCR. In *Populus* plants, identification of reliable reference genes has been carried out for qRT-PCR normalization in adventitious rooting of hardwood cuttings [3], in different tissues under long-day, short-day, or short-day plus low-temperature conditions and in different abiotic stress treatments [33, 34]. *Populus* is a woody perennial plant, which can undergo considerable secondary growth and form more wood each growing season. With the sequencing of its genome, *Populus* has become the model species for studies of the molecular mechanisms underlying secondary growth in wood formation. Specifically, the vertical stem segments of *Populus* can be used as a good system to study the molecular changes associated with secondary growth during wood formation since they represent different developmental stages from primary to secondary growth (Fig 1), which had been previously reported [4, 35, 36]. To the best of our knowledge, this is the first report carried out specifically to assess the expression stability of candidate reference genes in different stem segments in *Populus*, which represent primary growth, transition from primary to secondary growth, and secondary growth, respectively.

The reference genes that showed stable expression levels tended to be related to cell maintenance such as actin, tubulin, elongation factor and 18S ribosomal RNA. In this study, eight housekeeping genes were chosen for validation of the stably expressed reference genes. According to Zhang, the actin gene family in *Populus* contains eight genes [37]. Here *PtrACT6* was selected for assessment of the stable reference genes across the stem segments with different developmental stages, since this gene had comparable expression in bark, phloem, cambium, and xylem. The 18S rRNA transcripts were much more abundant than the others, as indicated by the lowest Cq value with 100 times dilution of cDNA template in this experiment and supported by other reports [33, 34, 38]. Moreover, the rRNA fraction in total RNA may quite differ from sample to sample [38]. In this way, there are drawbacks of using 18S rRNA as reference gene in qRT-PCR assay.

Previous research has shown that geNorm, BestKeeper, and NormFinder can produce results on final ranking of the suitable reference genes with minor to substantial discrepancies [3, 10, 39]. However, this study showed that all the three programs ranked the same four genes, *eIF5A*, *ACT6*, *EF1-beta* and *HIS3*, as the most stable, although the order differed somewhat. Meanwhile, reference genes *TUBB* and *RP* were identified to be the least stable genes by all the three programs (Table 3). Furthermore, both programs geNorm and BestKeeper suggested that the genes *eIF5A*, *ACT6*, and *EF1-beta* as together suitable stable reference genes, and that the combination of those three genes would be optimal for calculating the normalization factor (Fig 3 and Table 5). In summary, these results indicate that the three most stable reference genes, *eIF5A*, *ACT6*, and *EF1-beta* could be used for accurate normalization of a target gene in *P*. *tomentosa* stem development, especially for those genes involved in primary and secondary growth.

## Conclusions

This is the first study specifically designed to validate a set of candidate reference genes for gene expression normalization using qRT-PCR in *Populus* stems for both primary and secondary growth. In the present study, 8 candidate reference genes were evaluated for stable expression across a set of 22 samples using the programs geNorm, NormFinder, and BestKeeper. Analysis indicated that the combination of the three most appropriate reference genes, *eIF5A*, *ACT6*, and *EF1-beta*, can provide more accurate and reliable normalization of qRT–PCR analysis of target gene expression during the stem development leading to wood formation.
